# Is genetic counseling a stressful event?

**DOI:** 10.3109/0284186X.2011.604343

**Published:** 2011-08-24

**Authors:** Karin Nordin, Afsaneh Roshanai, Cathrine Bjorvatn, Katharina Wollf, Ellen M Mikkelsen, Ingvar Bjelland, Gerd Kvale

**Affiliations:** 1 Department of Public Health and Caring Sciences, Uppsala University, Uppsala, Sweden; 2 Department of Public Health and Primary Health Care, University of Bergen, Norway; 3 Department of Psychosocial Science, University of Bergen, Norway; 4 Department of Clinical Epidemiology, Aarhus University Hospital, Aarhus, Denmark; 5 Department of Clinical Psychology, University of Bergen, Norway

## Abstract

*Purpose*. The aim of this paper was to investigate whether cancer genetic counseling could be considered as a stressful event and associated with more anxiety and/or depression compared to other cancer-related events for instance attending mammography screening or receiving a cancer diagnosis. *Methods*. A total of 4911 individuals from three Scandinavian countries were included in the study. Data was collected from individuals who had attended either cancer genetic counseling (self-referred and physician-referred) or routine mammography screening, were recalled for a second mammograpy due to a suspicious mammogram, had received a cancer diagnosis or had received medical follow-up after a breast cancer-surgery. Data from the genetic counseling group was also compared to normative data. Participants filled in the Hospital Anxiety and Depression Scale twice: prior to a potentially stressful event and 14 days after the event. *Results*. Pre-counseling cancer genetic counselees reported significant lower level of anxiety compared to the cancer-related group, but higher levels of anxiety compared to the general population. Furthermore, the level of depression observed within the genetic counseling group was lower compared to other participants. Post-event there was no significant difference in anxiety levels between the cancer genetic counselees and all other groups; however, the level of depression reported in the self-referred group was significantly lower than observed in all other groups. Notably, the level of anxiety and depression had decreased significantly from pre-to post-events within the genetic counseling group. In the cancer-related group only the level of anxiety had decreased significantly post-event. *Conclusion*. Individuals who attend cancer genetic counseling do not suffer more anxiety or depression compared to all other cancer-related groups. However, some counselees might need additional sessions and extended support. Thus, identifying extremely worried individuals who need more support, and allocating further resources to their care, seems to be more sufficient.

Modern gene technology has made it possible to identify individuals at an increased risk for genetic diseases. Therefore, since increasing numbers of individuals will now desire access to information about their genetic risk for developing a disease, genetic counseling services have become an integral part of specialized health care system. An implicit assumption has been that receiving information about a potentially life-threatening event, such as a cancer diagnosis, may cause psychological and emotional difficulties, and that genetic counseling may facilitate adjustment to this information [1]. Nevertheless, information is required regarding the psychosocial impact of genetic services upon the individual, families and society in general.

It is anticipated that in the near future, extensive information regarding the genetic transmission of a number of diseases will be available and consequently genetic counseling will have a substantial impact on both the structure and the costs of health services. Thus, it seems reasonable that aside from wondering whether genetic counseling actually facilitates an individuals' adjustment and wellbeing, question whether there is evidence to indicate that genetic information may leads to significant increases in personal distress (anxiety and/or depression).

During the last decade, several systematic reviews documenting the psychological impact of learning of ones' genetic risk for developing certain illnesses have been performed. One of the main conclusions from these reviews is that genetic counseling does not have any harmful effect on counselees and is effective in reducing pre-counseling levels of anxiety, depression and distress [2-6]. In a Swedish study examining the effects of pre-symptomatic testing for breast/ovarian and colon cancer susceptibility genes [7] the levels of anxiety and depression among mutation carriers were compared to both non-carriers and a normative Swedish sample. In line with results recently published by our research group [8], the results of the study by Arver et al. [7] indicated that the carriers of the cancer gene had an equal level of anxiety compared to both non-carriers and the normative sample. In that study, women being tested for breast cancer genes filled in a questionnaire at the time of blood sampling prior to the genetic counseling session and were reported to have lower levels of depression compared to the normative controls. In addition, as in the majority of previous studies [2,8-10], the results indicated a decrease in the mean level of anxiety over time. In a recent review on the psychological impact of genetic testing on breast cancer patients [6], eight papers published between 1995 and 2004 were identified: all indicated that genetic counseling was not accompanied by any increase in anxiety or depression. However, none of the papers included in the Sclich-Bakker review [11-18], compared participants level of distress to normal controls. Moreover, controlled studies mostly compare the effect of different types of interventions on study groups or compare the levels of anxiety, depression or distress in one group over time [2]. Accordingly, comparing the impact of genetic counseling on anxiety and depression to other potential stressful events, together with comparisons between counselees and both the general population and other groups within the health care system, seems to be both necessary and logical.

The main aim of this paper was to study whether genetic counseling could be considered as a stressful event by the individuals who attend such sessions. An additional aim was to explore whether counselees were more anxious and/or depressed in comparison to other large cancer-related groups within the health care systems (e.g. individuals who are referred for mammography or those who are treated for cancer diseases).

The rationale was that if genetic counseling attendees were found to be more anxious or depressed, then there would be need for psychological interventions to explore these emotions in depth. Such evidence could then be taken into account when planning and allocating resources to the future care of individuals who demand information about their genetic risk for a particular disease.

## Participants and methods

In the current study, comparisons were made between the level of anxiety and depression among individuals who attended genetic counseling due to having an increased risk for developing cancer; individuals who attended mammography screening for non-hereditary cancer; various cancer patients and a random sample of the general population.

Data were collected from Sweden, Norway and Denmark, and a total sample of 4911 participants was included in the study. Participants from Norway were “self-referred” to genetic counseling, whilst in Sweden and Denmark participants were referred to genetic counseling by their primary health care physicians. This differences in referral method presented the opportunity to compare individuals who sought genetic counseling in order to receive a risk evaluation as a first step (comparable to primary care), to those individuals who were referred to genetic counseling by a primary health care physician since they were considered to be at an increased risk for developing cancer.

### Standard genetic counseling session

In current study, participants attended their first genetic counseling session. During the first counseling session, the counselor usually provides information regarding the differences between sporadic cancer versus hereditary cancer, basic genetics, and the risk of developing cancer as a carrier of a mutated gene. In addition, the geneticist estimates the risk for non-affected counselees' or for affected counselees' close relatives and supplies information about genetic testing and surveillance programs. Counselees who undergo genetic testing will attend an additional session in association with the disclosure of the test results.

All participants gave informed consent and the study was approved by the Norwegian and Swedish Ethical Committee, Danish National Board of Health and the Danish Data Protection Agency.

The term “stressful events” referred to: attending genetic counseling, attending a routine mammography, being recalled for another mammography appointment due to a previous suspicious mammogram, receiving a cancer diagnosis and going for a medical follow-up following surgery for cancer. The different groups and events are described in more detail both below and in Table I.

**Table I:** Participants' gender, age and available pre-and post-event HADS*

Population	N	Female gender	Age Mean (range)	Pre-event HADS N	Post-event HADS N
Genetic counselling					
Self-referred	221	81%	43 (18-78)	213	186
Physician-referred	674	96%	42 (16-80)	655	216
Cancer-related group					
Routine mammography	417	100%	55 (26-76)	415	0
Recalled after mammography (suspected cancer)	509	100%	54 (40-74)	508	0
Cancer diagnosis	527	61%	64 (32-91)	149	484
Medical follow-up after	45	100%	44 (19-52)		
breast cancer surgery				45	42
General population	2483	89%	52 (25-84)	2394	2394
Total	4911	89%	52 (16-91)		

* HADS: Hospital Anxiety and Depression Scale.

To analyze the data, the sample was divided in three groups; a genetic counseling group, a cancer-related group and the general population group. Data for each group is presented separately. Comparisons were also made between subgroups of genetic counseling and cancer-related groups. Follow-up data (14 days after the event) was not available for all participants.

## Study groups and events

### The genetic counseling group

#### Counselees referred to the genetic counseling by a physician

Participants were recruited between September 2003 and September 2004 from four genetic outpatient clinics at University Hospitals throughout Denmark, and one genetic clinic in Sweden over a period of six years (1999 to 2005).The criteria for inclusion in the study were an age above 18 years together with at least one first or second degree relatives having been diagnosed with breast, breast/ovarian or colorectal cancer. Counselees were referred to the genetic counseling by a general physician or a specialist due to being at an increased risk for developing a hereditary cancer. A consecutive sample of 674 counselees was included in this group and this consisted of 431 participants from Denmark and 243 participants from Sweden. In Denmark 76% of counselees who were asked to participate in the study accepted whilst in Sweden 89% of counselees agreed to participate. Follow-up data was only available for the Swedish counselees (n = 216; 84%, see Table I).

#### Self-referred counselees

This group consisted of individuals who had sought genetic counseling on their own initiative. A total of 275 counselees from three genetic out-patient clinics at University Hospitals in Norway (Bergen, Trondheim and Stavanger) were asked to participate in the study during 2003. Inclusion criteria were as described above. A total of 221 counselees (80%) agreed to participate in the study. The majority (65%) had a history of breast- and/or ovarian cancer, 23% had a history of colorectal cancer and 12% had a history of both cancer forms within their families.

### The cancer-related group

#### The routine mammography screening group

A total of 689 women attending for routine mammography screening at two hospitals in Denmark throughout 2003 and 2004 were asked to participate in the study. In total, 61% (n = 417) agreed to participate and filled out the questionnaire.

#### Women recalled for further examination due to suspicious mammograms

In 1997, 26086 women attended a population-based mammography screening program in Uppsala, Sweden. In total, 901 women (3.5%) were then recalled for further investigation due to a suspect result, for instance a lump in the breast. All 901 women were asked to participate in the study, of which 509 (56%) agreed to and were included.

#### Cancer patients

Between October 1993 and December 1995, a consecutive group of Swedish patients (n = 729), that were either newly diagnosed or under investigation for breast cancer (n = 331); colorectal cancer (n = 154); gastric cancer (n = 47) or prostate cancer (n = 197), were asked to participate in the study. Seventy-two percent of patients (n = 527) accepted and agreed to participate in the study. A subgroup of breast cancer patients (n = 149) had filled out the questionnaire before their diagnosis was confirmed. This created a unique opportunity whereby the level of anxiety and depression prior to diagnosis could also be assessed. This is often impossible since patients are generally included in studies only after having been diagnosed with cancer.

#### The medical follow-up group

During 2000 and 2001 a group of 72 women diagnosed with breast cancer in Sweden, were asked to participate in the study in association with their first medical follow-up after the cancer surgery. In total, 63% (n = 45) accepted and were included in the study. The only criterion for inclusion was being younger than 50 years of age at the time of diagnosis. This criterion was chosen due to a minor increased risk for hereditary cancer, but not so high a risk level that makes genetic counseling necessary.

#### The general population group

Normative data on anxiety and depression, using The Hospital Anxiety and Depression Scale (HADS), were obtained from the Nord-Trondelag Health Study (The HUNT-2 Study) [19]. The HUNT-2 study, one of the largest health studies ever conducted, was carried out between 1995 and 1997 with a focus on evaluating the medical history of individual. Consequently, this study now provides a unique database of personal and family medical histories. Of 92 936 eligible individuals, 66 140 (71.2%) participated in the HUNT-2 study [19]. Nord-Trondelag is one of the 19 counties in Norway and comprises 3% of the national population. Notably, the county is fairly representative of Norway as a whole except for a slightly lower mean level of education. Based on the population register, all inhabitants in the county aged ≥20 years were invited to participate. Data collection was performed using postal questionnaires and a clinical examination. A random sample of 2483 subjects, from the HUNT-2 study with the same proportion of male/ female and the same age range as in the clinical sample, was included in the present study.

### Instrument

The Hospital Anxiety and Depression Scale (HADS) [20] consisting of a seven-item subscale for measuring anxiety (HADS-A) and a seven-item subscale for measuring depression (HADS-D), was the instrument chosen to assess symptoms of anxiety and depression within this study. Each item has a choice of four responses with scores ranging from 0 (no symptoms) to 3 (maximum symptoms). The subscale scores range from 0 to 21. The HADS is extensively used and has been demonstrated to possess good psychometric properties for use within both the normal population, and in somatic, psychiatric, and primary care patients [21].

### Procedure

Approximately half of the sample, 2428/4911 individuals filled out the HADS in connection with a potential stress-full event 14 days before the event and 14 days after the event. This data was compared to the data from a random sample of the general population (n = 2483).The proportion of men (11%) and women (89%) were equal in both samples.

### Statistical analysis

Comparisons of the mean values were performed by unpaired two-tailed t-tests. Pre to post-event changes were investigated by paired t-tests. T-tests were used instead of two-way ANOVA due to lack of follow-up data for most participants. Analyses of variance (ANOVA) were used for comparison of three or more groups. Due to unequal sample sizes, post hoc comparisons were performed with the Tukey's Honestly Significantly Different (HSD) Unequal Sample Sizes test. Post hoc test results are reported only for comparisons between genetic counseling group (as a whole or as self-referred and physician-referred subgroups) and each of the other included groups.

## Results

### Between-group comparisons of anxiety and depression

#### Pre-event anxiety and depression

Prior to the stress-full event, the genetic counseling group reported significantly lower levels of anxiety (M = 5.6), compared to the cancer-related group (M = 6.9), but higher levels of anxiety compared to the general population (M = 4.4) [F (2.61) = 223.3, p < 0.001] (Table II). However, the level of depression (M = 2.7) reported by the genetic counseling group was lower compared to both the cancer-related group (M = 3.4) and the general population (M = 3.5), [F (2.62) = 31.9, p < 0.001] (Table II).

**Table II:** Participants' mean level of anxiety and depression.

Population	Pre-event anxiety Mean (SD)	Post-event anxiety Mean (SD)	Pre-event depression Mean (SD)	Post-event depression Mean (SD)
Genetic counseling group	5.6 (4.0)	5.0 (4.1)	2.7 (3.0)	2.3 (2.9)
Cancer-related group	6.9 (4.5)	5.1 (4.5)	3.4 (3.2)	3.8 (3.7)
General population	4.4 (3.4)	4.4 (3.4)	3.5 (3.1)	3.5 (3.1)
F	223.3*	17.3*	31.9*	57.3*

Note: values for normal population were collected once, not pre and post.

* p < 0.001.

**Table III:** Comparison of the level of anxiety and depression in genetic counseling subgroups pre- and post-event.

Population	Pre-event anxiety Mean (SD)	Post-event anxiety Mean (SD)	Pre-event depression Mean (SD)	Post-event depression Mean (SD)
Genetic counseling group				
A- self-referred	4.9 (3.6)	4.4 (3.9)	2.7 (2.9)	2.5 (3.1)
B- physician-referred	5.8 (4.1)	5.4 (4.3)	2.6 (3.0)	2.7 (2.9)
Comparison A-B	t = 2.9^a^	t = 2.4^b^	t = 0.01	t = 0.1

p < 0.001.

p < 0.05.

#### Post-event anxiety and depression

After counseling, no differences in anxiety were observed between the genetic counseling group (M = 5.0, SD = 4.1) and the other groups [F (2.54) = 17.3, p < 0.001)] (Table II).

The genetic counseling group reported significantly lower levels of depression (M = 2.3, SD = 2.9) compared to both the cancer-related group and general population (M _cancer-related_ = 3.8, SD = 3.7, M_general population_ = 3.5, SD = 3.1) [F(2.55) = 57.3, p < 0.001] (Table II).

### Between-subgroup comparisons of anxiety and depression

The self-referred counselees reported lower levels of anxiety both before (M = 4.9, SD = 3.6), and after (M = 4.4, SD = 3.9) the counseling session compared to the physician-referred counselees (M _pre-counseling_ = 5.8, SD = 4.1, M _post-counseling_ = 5.4, SD = 4.3). There was no corresponding difference for depression between the two groups (Table IV).

**Table IVa:** Comparison of the level of anxiety and depression in self-referred counselees with the other included groups.

Population	Pre-event anxiety Mean (SD)	Post-event anxiety Mean (SD)	Pre-event depression Mean (SD)	Post-event depression Mean (SD)
Self-referred counselees	4.9 (3.6)	4.4 (3.9)	2.7 (2.9)	2.5 (3.1)
Routine mammography	5.9 (4.1)^a^		2.9 (3.1)	
Recall after mammography (cancer suspicion)	7.7 (4.5)^b^		3.5 (3.1)^a^	
Cancer diagnosis	7.0 (4.9)^b^	5.0 (4.5)	3.9 (3.9)^b^	3.8 (3.7)^b^
Medical follow-up after cancer surgery	6.3 (4.3)	6.0 (4.2)	4.3 (3.5)^a^	4.1 (3.6)^a^
General population	4.5 (3.5)	4.5 (3.5)	3.5 (3.1)^b^	3.5 (3.1)^b^

a p ≤ 0.05.

b p ≤ 0.01.

Prior to the counseling session, both genetic counseling subgroups reported lower levels of anxiety compared to most of the other cancer-related subgroups and the physician-referred group reported higher level of anxiety compared to the general population [F(6.43) = 57.3, p < 0.001]. No significant differences in anxiety were observed between the genetic counseling and the other included groups post-counseling [F (3.924) = 2.5, P > 0.05] (Table IVa and b).

**Table IVb:** Comparison of the level of anxiety and depression in physician-referred counselees with the other included groups.

Population	Pre-event anxiety Mean (SD)	Post-event anxiety Mean (SD)	Pre-event depression Mean (SD)	Post-event depression Mean (SD)
Physician-referred genetic counselees	5.8 (4.1)	5.4 (4.3)	2.6 (3.0)	2.7 (2.9)
Routine mammography	5.9 (4.1)		2.9 (3.1)	
Recall after mammography (cancer suspicion)	7.7 (4.5)^b^		3.5 (3.1)^b^	
Cancer diagnosis	7.0 (4.9)^a^	5.0 (4.5)	3.9 (3.9)^b^	3.8 (3.7)^b^
Medical follow-up after cancer surgery	6.3 (4.4)	6.0 (4.2)	4.3 (3.5)^b^	4.1 (3.6)
General population	4.5 (3.5)^b^	4.5 (3.5)	3.5 (3.1)^b^	3.5 (3.1)^b^

a p ≤ 0.05.

b p ≤ 0.01.

The genetic counseling subgroups separately reported significantly lower levels of depression both pre [F (6.44) = 11.9,p < 0.001] and post-counseling [F(3924) = 9.9, p < 0.001] compared to most other groups (Table IVa and b). The only exception pre-event was that no significant difference was reported by those attending for routine mammography and post-event no significant difference was reported between the physician-referred counselees and the medical follow-up group (Table IVa and b).

### Within-group comparisons of anxiety and depression (changes over time)

Anxiety levels decreased significantly from pre-to-post-event measurement in both the genetic counseling group [M_pre-event_ = 5.5, M_post-evem_ = 5.0, (t = 3.7, p < 0.001)], and the cancer-related group [M_pre-event_ = 6.7, M_post-event_ = 5.2, SD = 4.7, (t = 4.6, p < 0.001)] (Table V).

**Table V:** Within-group comparisons of the level of anxiety and depression over time (paired t-test)^a^.

Population	Pre-event anxiety Mean (SD)	Post-event anxiety Mean (SD)	t-value	Pre-event depression Mean (SD)	Post-event depression Mean (SD)	t-value
Genetic counseling group	5.5 (4.1)	5.0 (4.2)	3.7*	2.9 (3.0)	2.6 (3.0)	3.1*
Cancer-related group	6.7 (4.7)	5.2 (4.7)	4.6*	3.8 (3.7)	3.6 (3.8)	1.1

a Note that different mean values (compared to the Table III) are due to the different number of participant pre to post-event.

* p < 0.001.

Furthermore, the level of depression decreased significantly in the genetic counseling group from pre-to post- event measurement [M _pre-event_ = 2.9, M_Post-event_ = 2.6, (t = 3.1, p < 0.001)]. However, no significant decrease in the level of depression was observed in the cancer-related group [M _Pre-event_ = 3.8, M_post-event_ = 3.6, (t = 1.1, p > 0-05)] (Table V).

### Within-subgroup comparisons of anxiety and depression (changes over time)

The results from analyzing changes in the level of both anxiety and depression over time within each subgroup indicated that only physician-referred counselees reported a significant reduction in the level of anxiety [M_pre-event_ = 6.0, M_post-event_ = 5.4, t = 3.6, p < 0.001] and depression [M_pre-event_ = 3.2, M_Post-event_ = 2.7, (t = 3.9, p < 0.001)] over time (Table VI). The only other subgroup who reported a significant decrease in anxiety post-event was the cancer diagnosis group [M_pre-event_ = 7.0,M_post-event_ = 5.0 (t = 5.1, p < 0.001)] (Table VI).

**Table VI:** Within-subgroup comparisons of the level of anxiety and depression over time (paired t-test)^a^.

Population	Pre-event anxiety Mean (SD)	Post-event anxiety Mean (SD)	t	Pre-event depression Mean (SD)	Post-event depression Mean (SD)	t
Genetic counselling self-referred	4.9 (3.6)	4.6 (3.9)	1.7	2.6 (2.8)	2.5 (3.1)	0.6
Physician-referred	6.0 (4.4)	5.4 (4.3)	3.6	3.2 (3.2)	2.7 (2.9)	3.9*
Cancer diagnosis	7.0 (4.9)	5.0 (4.5)	5.1	3.8 (3.9)	3.9 (3.7)	1.3
Medical follow-up after cancer surgery	5.7 (4.0)	5.8 (4.0)	0.3	3.9 (3.2)	3.9 (3.5)	0.3
General population^b^	4.5 (3.5)	4.5 (3.5)		3.5 (3.1)	3.5 (3.1)	

Note that different mean values (compared to the Table III) are due to the different number of participant pre to post-event.

Values for normal population is collected only once, not pre-and post-event.

* p < 0.001.

## Discussion

Based on the results of this study, before attending counseling, individuals within the genetic counseling group, reported significantly lower level of anxiety and depression compared to those in the cancer-related group and higher levels of anxiety and lower level of depression compared to the general population. Post-event, there was no difference in the level of anxiety between the genetic counseling group and the cancer-related group, but the level of depression was still lower than both the cancer-related group and the general population.

Compared to the general population, the cancer-related group still reported a higher level of anxiety and depression post-event.

The level of anxiety and depression had decreased significantly in the genetic counseling group from pre-to-post-event measurement. However, this lower level of anxiety and depression was only observed in the counselees who were referred to the genetic counseling by a physician. The other group who reported a significant decrease in anxiety post-event was the cancer diagnosis group.

The significantly lower level of anxiety and depression reported by the physician-referred counselees post-counseling supports the important role played by genetic counseling.

Individuals probably experienced high levels of unnecessary distress prior to counseling, and attendance at the counseling session may have helped them to realize the actual risk and the possibility of receiving further help.

It was not surprising that the women who were recalled for further mammography reported the highest levels of anxiety, since uncertainty and being at risk of receiving a cancer diagnosis may be considered as a very stressful event [22].

Depending on whether genetic counseling is primarily considered as a session for delivering precise information regarding the genetic risk for a given illness [23], or is considered in terms of its psychosocial elements [24] leads to different conclusions with respect to the care to be offered [25].

Genetic counseling is considered as a psycho-educative process due to the notion that genetic information might give rise to a substantial amount of psychological distress [1,26,27], and attending genetic counseling, as was observed in the current study, might reduce any distress experienced [28].

In line with previous studies [9,29,30], our results clearly demonstrated that attending genetic counseling, whether self-referred or following a physician's recommendation, could be related to some distress, but was not associated with higher levels of anxiety and depression compared to the other kinds of events related to the possibility of having cancer (e.g. being recalled to mammography), or actually receiving a cancer diagnosis. In fact they reported even lower levels of anxiety and depression. Furthermore, the level of anxiety and depression had decreased significantly in the genetic counseling group from pre-to-post-event measurement and this may confirm that any distress experienced is temporary. Also, individuals confronted with these types of events (attending genetic counseling, and mammography or receiving a cancer diagnosis) experience higher levels of anxiety compared to the general population, but as was observed in previous studies [31,32] this elevation is transient: seeing the doctor or counselor leads to a significant reduction in anxiety.

As mentioned earlier, anxiety levels in counselees who seek genetic counseling on their own initiative was, pre-counseling, significantly lower than counselees referred to the genetic counseling by a physician, and compared to most other studied events. This is not unexpected since having a suspicious mammogram, receiving a cancer diagnosis or being operated for breast cancer might be associated with higher level of anxiety. Nevertheless, it is possible that the lack of significance in the group of self-referred counselees could be due to the smaller size of this group or because physicians refer individuals who have higher risk of developing cancer and are more anxious to the counseling to help them to receive realistic risk estimation.

It is noteworthy that at the time of collecting data for the current study, genetic counseling was a relatively new activity. It seems reasonable to speculate that this first wave of individuals who sought counseling on their own probably were more motivated, had more information about cancer and perhaps were not as anxious or depressed as individuals who are referred by another person. As the genetic risk evaluation will be used more widely it is reasonable to assume that individuals who seek genetic counseling might not have the same characteristics and will require a different type of care.

In accordance with an earlier report [7], our results indicated that individuals attending genetic counseling were significantly less depressed before and after the counseling compared to other patients, i.e. those who were facing the possibility of receiving a cancer diagnosis, those who actually had received a diagnosis, and to the general population. This result could be due to the fact that individuals who seek genetic counseling are actually a selective group and posses certain characteristics that were mentioned earlier. This also might apply to those who are referred by a physician. A considerable proportion of the counselees had high education status, were cohabitant individuals who probably have a better socioeconomic position, no economical worries, plenty of information and a better social network which can provide good support. In addition, it is reasonable to assume that at-risk individuals who have a higher level of distress do not attend genetic counseling.

### Methodological considerations

One of the most important strengths of the current study is that the results are based on a very large sample from different clinics from three Scandinavian countries. Furthermore, the study had two unique advantages. The first was the possibility to compare self-referred counselees, who probably have different characteristics, to individuals who are referred to genetic counseling by a physician due to being at an increased risk for developing cancer. The second was the opportunity of having access to data for a subgroup of breast cancer patients before their diagnosis was confirmed. This made it possible to assess the level of anxiety and depression pre-diagnosis possible; a variable which is often impossible to measure since patients are usually included in studies only after having been diagnosed by cancer.

In addition, psychological distress was assessed with a well established instrument proven to have good psychometric properties.

However, the study is not without limitations and the results need to be considered in the light of these short-comings. For instance, the homogeneity of the groups was not optimal and the date of measurement was different for various subgroups. However, the time points of pre to post-measurements had been the same for all groups.

The representativeness of women recalled for further examination (response rate 56%) may be doubtful and generalization of these results should be done with caution due to the selectiveness of the sample. The lack of follow-up data is also an undesirable fact which may have affected the results.

## Conclusion

Genetic counseling can give rise to some distress, though it does not seem to be a more stressful event compared to other events within the health care service. On the contrary, genetic counseling may be associated with some transient elevations of anxiety that for most individuals will be handled without the requirements for any specific intervention.

Considering these results and the findings of previous studies indicating that the level of distress in genetic counseling counselees decreases over time, one plausible conclusion might be that genetic counseling probably helps counselees to cope with their cancer or cancer susceptibility over the long term. However, some counselees might need additional sessions and extended support. Thus, it seems that identifying individuals who are extremely worried and need more support, and allocating additional resources to the care of them, is more important.

## NOTICE OF CORRECTION

The Early Online version of this article published online ahead of print on 24th of August 2011 contained an error on page 1. The first author was placed as second author. This has been corrected for the current version.
